# Bovine milk microbiota: Evaluation of different DNA extraction protocols for challenging samples

**DOI:** 10.1002/mbo3.1275

**Published:** 2022-03-31

**Authors:** Julia A. Schwenker, Meike Friedrichsen, Silvio Waschina, Corinna Bang, Andre Franke, Ricarda Mayer, Christina S. Hölzel

**Affiliations:** ^1^ Department for Animal Hygiene and Health, Institute of Animal Breeding and Husbandry Christian‐Albrechts‐University Kiel Germany; ^2^ Institute of Human Nutrition and Food Science, Nutriinformatics Christian‐Albrechts‐University Kiel Germany; ^3^ Institute of Clinical Molecular Biology Christian‐Albrechts‐University Kiel Germany; ^4^ Department of Veterinary Sciences Ludwig‐Maximilians‐University Munich Oberschleißheim Germany; ^5^ GNA Biosolutions GmbH Martinsried Germany

**Keywords:** bovine milk, DNA extraction, Illumina amplicon sequencing, milk microbiome

## Abstract

The use of an adequate protocol that accurately extracts microbial DNA from bovine milk samples is of importance for downstream analysis such as 16S ribosomal RNA gene sequencing. Although sequencing platforms such as Illumina are very common, there are reservations concerning reproducibility in challenging samples that combine low bacterial loads with high amounts of host DNA. The objective of this study was to evaluate six different DNA extraction protocols applied to four different prototype milk samples (low/high level of colony‐forming units [cfu] and somatic cells). DNA extracts were sequenced on Illumina MiSeq with primers for the hypervariable regions V1V2 and V3V4. Different protocols were evaluated by analyzing the yield and purity of DNA extracts and the number of clean reads after sequencing. Three protocols with the highest median number of clean reads were selected. To assess reproducibility, these extraction replicates were resequenced in triplicates (*n* = 120). The most reproducible results for α‐ and β‐diversity were obtained with the modified DNeasy Blood & Tissue kit after a chemical pretreatment plus resuspension of the cream fraction. The unmodified QIAamp DNA Mini kit performed particularly weak in the sample representing unspecific mastitis. These results suggest that pretreatment in combination with the modified DNeasy Blood & Tissue kit is useful in extracting microbial DNA from challenging milk samples. To increase reproducibility, we recommend that duplicates, if not triplicates, should be sequenced. We showed that high counts of somatic cells challenged DNA extraction, which shapes the need to apply modified extraction protocols.

## Graphical abstract

This study evaluates six different DNA extraction protocols applied to four different prototype milk samples (low/high level of colony‐forming units and somatic cells). DNA extracts were amplified on Illumina MiSeq with primers for the hypervariable regions V1V2 and V3V4. We conclude that the different extraction protocols achieve various reproducible results in terms of α‐ and β‐diversity as well as read number, even on the replicate level of extraction and sequencing.

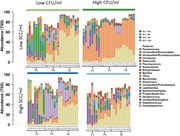

## 1 INTRODUCTION

Bovine mastitis is an inflammation of the mammary gland and is one of the most important factorial diseases in dairy herds (Oviedo‐Boyso et al., 2007). For ages, it was a common belief that the udder is primarily sterile. Microorganisms in a milk sample with low somatic cell count (SCC) were thought to occur mainly due to contamination. However, in recent years it has become apparent that the udder of dairy cows is endowed with a complex microbial community (Addis et al., 2016). New approaches allow to characterize the composition of the microbial community (Addis et al., 2016), and sequencing of 16S ribosomal RNA (rRNA) gene amplicons is now used to depict the microbial diversity of healthy quarter milk samples (Kuehn et al., 2013; Oikonomou et al., 2012), and also in case of mastitis (Andrews et al., 2019; Falentin et al., 2016; Lima et al., 2018). To deliver accurate and representative sequencing results, high‐quality DNA is a prerequisite (Burbach et al., 2015). According to Pirondini et al. (2010), it is a well‐known problem that suitable DNA extracts from milk are hard to obtain. This might be related to physical, chemical, and biological characteristics of milk, such as fat, protein, and calcium molecules as well as bacterial and mammalian debris (Wilson, 1997). Additionally, plasmins can also serve as natural inhibitors in milk that degrade *Taq* polymerases (Powell et al., 1994).

Challenges are even more complex if we aim to reproducibly extract complex communities from milk, since the structural properties of bacterial species can affect the DNA yield, thus leading to a falsification of the subsequent microbial analysis (Lima et al., 2018; Unno et al., 2015).

Different extraction protocols have been used in past studies on milk samples (Cremonesi et al., 2006; Lima et al., 2018; Quigley et al., 2012; Unno et al., 2015), combining the use of detergents with subsequent chemical or mechanical lysis. The use of mechanical lysis by bead beating has already been shown in several studies to be a very effective DNA extraction method from milk samples of different fractions (raw milk, cream and fat fraction, healthy and clinical mastitis samples; Bonsaglia et al., 2017; Ganda et al., 2016; Lima et al., 2018; Quigley et al., 2012). Besides the type of cell lysis, the importance of removing the fat layer is also discussed, as this is a common procedure of commercial kits (Hunt et al., 2011; Urbaniak et al., 2016). Often, only the pellet is used for further processing and the cream and whey fractions are discarded (Gao et al., 2007). It is also stated that lipid‐rich samples can affect DNA yield by affecting DNA disruption or the chemistry of the DNA isolation buffers (Macherey & Nagel, 2016). Gao et al. (2007), on the other hand, reported that bacteria equipped with a complex lipid‐rich cell wall system preferentially pass into the cream fraction. By using a cationic detergent and pooling pellet and cream fraction, the authors generated higher DNA yields of *Mycobacterium avium* subsp. *paratuberculosis*.

The objective of this study was to compare six different DNA extraction protocols in terms of their suitability to allow wide‐range, reproducible DNA‐extraction of complex microbial communities (“microbiome analysis”) from differently composed bovine milk. For this purpose, repeatability of DNA extraction and two amplicon sequencing approaches (V1V2 and V3V4) were assessed and the richness and diversity of bacterial communities in the samples were compared. Prototype samples included milk rich in somatic cells and bacteria as well as milk from inflamed, but uninfected and healthy glands since we hypothesized that protocols perform differently in these different conditions.

## 2 MATERIALS AND METHODS

### 2.1 Sample collection

Sampling was conducted on a research dairy farm of the Kiel University in Schleswig‐Holstein, Germany. The farm is mainly used for commercial dairy production. None of the cows was involved in invasive trials during the study or before study onset. None of the cows included in the trial was antibiotically treated within 12 weeks before sampling. For culture and microbiome analysis, quarter foremilk samples were aseptically collected from six Holstein‐Friesian cows in a rotary milking parlor (28 stalls; GEA Group) immediately before evening milking. Sampling was carried out according to the standard recommendations by the National Mastitis Council's Laboratory Handbook on bovine mastitis (National Mastitis Council, 2017). Briefly, teats were forestripped and thoroughly disinfected with wipes soaked in 70% ethanol. First milk streams were discarded and then 25 ml of quarter foremilk were collected in quadruplicate into sterile 50‐ml plastic tubes (CELLSTAR; Greiner Bio‐One). Separate quarter foremilk samples were taken to measure the SCC (cells/ml milk) using a Fossomatic 7 DC (Foss A/S). The samples were transported on ice and then homogenized by mixing them thoroughly at 2500 rpm for 5 min using a Vortex‐Genie 2 (Scientific Industries). After mixing, 1 ml milk was diluted at 1:10 in ¼ Ringer's solution (¼ strength). Undiluted and diluted milk (0.1 ml each) were plated on blood agar containing 5% sheep blood (Thermo Fisher Scientific) and incubated aerobically at 37°C for 24 h. Colonies were counted after 22 ± 2 h and recorded as colony‐forming units (cfu/ml milk). The microbial count was determined by averaging the counts of the 1:10‐diluted and the undiluted milk. Quarter milk samples were divided into 16 aliquots depending on the volume needed for the different protocols and stored at −20°C until further processing. Based on the analysis of cfu and SCC we excluded samples with signs of acute specific mastitis (high cfu, pure culture, somatic count >200,000 cells/ml). Finally, 4 out of 24 quarter milk samples were selected, applying a 2 × 2 design (Table 1). The data of all 24 quarter milk samples can be seen in Table A2. To classify the milk samples into “low” and “high” SCCs (SCC+SCC−), a threshold of 200,000 somatic cells per ml milk was set (Schukken et al., 2003). For this study, quarter milk samples with “low” cfu (cfu−) values represented samples below 100 cfu/ml. Samples with “high” cfu (cfu+) values exceeded a cfu value of 400 cfu/ml milk. These samples were prototypes for four different situations: sample 5_2 was unsuspicious of mastitis (SCC−cfu−), samples 4_2 and 3_1 resembled different situations of unspecific mastitis (SCC+cfu− and SCC+cfu+). The last sample 5_3 (SCC−cfu+) represented the situation found in latent mastitis or fresh infections or apathogenic colonization or after contamination (which was excluded here).

**Table 1:** SCC and cfu values of quarter milk samples (per ml milk) selected in a 2 × 2 design

	“Low” (cfu/ml)		“High” (cfu/ml)	
	Sample 5_2	65 cfu/ml	Sample 5_3	495 cfu/ml
“Low” SCC/ml		86,000 SCC/ml		117,000 SCC/ml
	Sample 4_2	90 cfu/ml	Sample 3_1	725 cfu/ml
“High” SCC/ml		318,000 SCC/ml		393,000 SCC/ml

Abbreviations: cfu, Colony‐forming unit; SCC, somatic cell count.

### 2.2 DNA extraction

Six different DNA extraction protocols, some of which have been used in previous studies (Gao et al., 2007; Husakova et al., 2020; Lima et al., 2018; Mayer, 2018; Oikonomou et al., 2012) were compared. The characteristics of the DNA extraction protocols are summarized in Table 2. The samples were thawed and thoroughly homogenized. The kits were run in triplicate, one run on the first day and two on the following day to account for daily variance. This did not apply to the modified QIAamp DNA Mini kit (P5) and the modified DNeasy Blood & Tissue kit (P4), which were only run in duplicate. Replicate extractions (three for each) were performed on two different days. In addition, a negative control sample (nuclease‐free water) was included in each extraction method and replicate (Invitrogen, Thermo Fisher Scientific).

**Table 2:** Brief description of the characteristics of the DNA extraction kits used in this study

Protocol no.	Extraction kit	Cell lysis	Principle
P1	DNeasy PowerSoil kit (Qiagen)	BB	Based on chaotropic agents, detergents, inhibitor removal technology, bead size 0.7 mm garnet, binding DNA on silica membrane spin column.
P2	DNeasy PowerFood Microbial kit (Qiagen)	BB	Based on chaotropic agents, detergents, inhibitor removal technology, bead size 0.15 mm garnet, binding DNA on silica membrane spin column.
P3	QIAamp DNA Mini kit (Qiagen)	CL	Based on chaotropic agents, proteinase K, and heating, binding DNA on silica membrane spin column.
P4	Modified DNeasy Blood & Tissue kit (Qiagen)	CL	Pretreatment before lysis: heating, cooling, resuspension of the cream fraction with a detergent, lysis with chaotropic agents, enzymes, reheating, binding DNA on silica membrane spin column.
P5	Modified QIAamp DNA Mini kit (Qiagen)	CL	Pretreatment before lysis: heating, cooling, resuspension of the cream fraction with a detergent, lysis with chaotropic agents, proteinase K, reheating, binding DNA on silica membrane spin column.
**P6**	Modified DNeasy Blood & Tissue kit (Qiagen)	CL	Lysis with enzymes and extended incubation times and additional boiling lysis, binding.
			DNA on silica membrane spin column.

Abbreviations: BB, bead beating; CL, chemical lysis.

DNA extraction started with a milk volume of 250 µl when using the DNeasy PowerSoil kit (P1, Qiagen) and with 1.5 ml initial milk volume for the DNeasy PowerFood Microbial kit (P2, Qiagen). Both protocols were performed according to the manufacturer's instructions without modifications. Briefly, both protocols are based on six steps: homogenization, mechanical and chemical lysis by bead beating, chaotropic agents and detergents, inhibitor removal, binding of the DNA to the silica membrane, wash process, and an elution step. The QIAamp DNA Mini kit (P3, Qiagen) was also performed according to the manufacturer's instructions without modifications. Here, we used the protocol for DNA purification from blood or body fluids (200 µl initial volume). Specifically, enzymatic lysis with 20 µl proteinase K was performed with incubation at 56°C, followed by the same wash and elution steps as for P1 and P2. The modified QIAamp DNA Mini kit (P5; Qiagen) and the modified DNeasy Blood & Tissue kit (P4; Qiagen) represented a modification (pretreatment before lysis) based on Gao et al. (2007). Ten milliliters of the initial volume of the quarter milk sample were heated for 10 min at 95°C in a water bath and then cooled in ice water for 10 min. After a centrifugation step at 3100*ɡ* for 30 min (4°C), the supernatant (without destroying the cream fraction) was removed using a cannula and disposable syringe. The cream fraction and pellet were then resuspended with 15 ml of freshly prepared 0.75% hexadecylpyridinium chloride (HPC) (Merck KGaA). Subsequently, the tubes were incubated at room temperature for 30 min by thoroughly mixing every 5 min. After the pretreatment with HPC, centrifugation was performed for 15 min (4°C) at 2000*ɡ*. Then, the supernatant including the cream fraction was discarded. The remaining steps were performed according to the manufacturer's instructions of the QIAamp DNA Mini Kit and the DNeasy Blood & Tissue kit including a recommended modification for Gram‐positive bacteria. Each protocol was finally eluted with 200 µl nuclease‐free water, except for the Power Food and Power Soil kit, where 100 µl was used. The modified DNeasy Blood & Tissue kit (P6; Qiagen) is a modification of the protocol for Gram‐positive bacteria with extended incubation times and additional boiling lysis. Ten milliliters of the initial volume were thoroughly mixed and centrifuged at 4000*ɡ* for 10 min (4°C). The obtained pellet was resuspended with 180 µl lysis buffer (20 mM Tris‐Cl, pH 8.0, 2 mM sodium EDTA, 1.2% Triton X‐100 [Merck KGaA], 20 mg/ml lysozyme with an enzymatic activity of ≥45,000 FIP U/mg [Thermo Fisher Scientific]). Subsequently, the pellet was incubated at 37°C in a water bath for 1.5 h. After the addition of 25 µl proteinase K and 200 µl buffer AL, it was further incubated for 1.5 h at 56°C, followed by subsequent boiling lysis for 10 min at 99°C. After cooling down to room temperature (~22°C), the remaining steps were performed according to the manufacturer's instructions. Two laboratory workers processed the extraction kits; one processed all replicate extractions of P1, P4, P5, and P6, whereas the second one performed all replicate extractions with P2 and P3.

### 2.3 DNA quality and quantity

DNA concentrations and purity of the extracts from milk and nuclease‐free water (control) were assessed using a NanoDrop ND‐1000 spectrophotometer (VWR International). According to the manufacturer's information, this device measures reliably up to a double‐stranded DNA concentration of 3700 ng/µl. To determine DNA concentrations, absorbance at wavelengths of 260 nm (*A*_260_) and 280 nm (*A*_280_) were measured. DNA purity was assessed based on the absorbance ratio 260/280 and 260/230 of each sample. Each sample was measured three times and the instrument was calibrated with nuclease‐free water. The instrument was blanked with the elution buffers supplied with each kit. Data of measured DNA yield and purity are shown in Table A1.

### 2.4 Illumina amplicon sequencing

Amplification of the V1V2 and the V3V4 hypervariable regions of the bacterial 16S rRNA gene was performed via one‐step polymerase chain reaction (PCR) using barcoded primers. The nucleotide sequences of the primer for the V1V2 hypervariable regions were as follows: 27F, 5′‐AGAGTTTGATCCTGGCTCAG‐3′; 338R, 5′‐TGCTGCCTCCCGTAGGAGT‐3′ (Hamady et al., 2008). Primers 341F (5′‐CCTACGGGAGGCAGCAG‐3′) and 806R (5′‐GGACTACHVGGGTWTCTAAT‐3′) were used for the amplification of the V3V4 region (Caporaso et al., 2011; Muyzer et al., 1993). A no‐template control (NTC) and a MOCK community DNA standard (Zymo Research Europe GmbH) with predetermined bacterial species were included (*Bacillus subtilis*, *Cryptococcus neoformans*, *Escherichia coli*, *Enterococcus faecalis*, *Listeria monocytogenes*, *Lactobacillus fermentum*, *Pseudomonas aeruginosa*, *Salmonella enterica*, *Staphylococcus aureus*, *Saccharomyces cerevisiae*). The reactions were performed using a mixture (15 µl) containing 0.15 µl Phusion Hot Start II Polymerase (2 U/µl) with 3 µl reaction buffer (Thermo Fisher Scientific), 10 mM dNTP mix, 10 µM of each primer, 8.95 µl H_2_O (Invitrogen, Thermo Fisher Scientific), and 2 µl of template DNA. Amplification steps consisted of the following: an initial denaturing step of 98°C for 3 min, which was followed by 30 cycles of 98°C for 9 s, 50°C for 1 min, 72°C for 1 s, and an elongation step for 72°C for 10 min. PCR products were then purified and normalized with the SequalPrep Normalization Plate kit (Thermo Fisher Scientific) according to the manufacturer's guidelines. In total, 80 samples (plus NTC and MOCK) were submitted for sequencing. Based on the number of reads three DNA extraction protocols were selected for subsequent sequencing in triplicate. Protocols were selected for this second step if sequencing with both primer pairs reached median clean‐read numbers above 1000. Subsequently, PCR was performed in triplicate for each of the original DNA‐extract triplicates or duplicates. Final equimolar libraries were sequenced using the paired‐end MiSeq reagent kit v3 (2 × 300 bp chemistry) on the MiSeq platform (Illumina Inc.) according to Caporaso et al. (2012).

### 2.5 Bioinformatic analysis

Microbiome data processing was performed using the DADA2 version 1.10 workflow for big data sets (Callahan et al., 2016) resulting in abundance tables of amplicon sequence variants (ASVs). Briefly, sequences were handled separately and finally collected in a single abundance table per data set and checked for chimeras. ASVs underwent taxonomic annotation using the naïve Bayesian classifier provided in DADA2 and using the Ribosomal Database Project (Wang et al., 2007). Data were quality checked and obvious contaminants were removed in further steps of the analysis (see Appendix A). ASVs that appeared only in water extractions but not in milk were deleted from the data set. Water extractions were checked for ASVs appearing in more than two replicates with at least once >100 reads. These ASVs were left in the data set but flagged for review after analysis of results. Analyses and visualization of ASV data were generated with Calypso version 8.84 (Zakrzewski et al., 2017). Samples with less than 500 sequence reads and taxa that had less than 0.01% relative abundance across all samples were removed by applying the respective filter options in Calypso. The selection of taxa was set to the maximum value (20,000 taxa). Data were normalized to relative abundance using total sum scaling (TSS), a method for normalizing count data by dividing feature read counts by the total number of reads in each sample. Statistical analysis and data visualization was performed using Calypso software. Clean reads were depicted in boxplots showing the minimum, maximum, median, and mean values. The relative abundance of the top 20 genera was comparatively depicted in pie charts. Repeatability was assessed by (i) comparing numbers of clean reads between extraction and sequencing replicates, (ii) comparing the variability of α‐diversity indices between replicates, (iii) a cluster analysis, and (iv) a principal coordinate analysis (PCoA, see below). An analysis of variance was applied to diversity indices (Shannon, richness, evenness), of the 2 × 3 (P4) to 3 × 3 (P3, P6) replicates of each prototype sample × kit combination, to test differences in α‐diversity between (i) samples and (ii) the applied DNA extraction protocols. β‐Diversity was estimated using Bray–Curtis distance matrices and visualized by plotting the two main coordinate axes obtained from the PCoA. The α‐ and β‐diversity was evaluated protocol‐ and primer‐specific, as we wanted to focus on the performance of each protocol when applied to different kinds of samples (see also Section 4).

## 3 RESULTS

### 3.1 Pretrial

**Figure 1:**
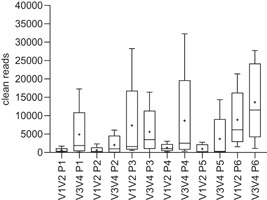
Clean reads of milk samples sequencing (V1V2 and V3V4) after applying different DNA extraction protocols (P1–P6, for description see Table 2). All samples (*n* = 4) were extracted in duplicate (P4 and P5) or in triplicate (P1, P2, P3, and P6). MOCK standard and NTC had clean reads of 21,812 and 545 in the V1V2 run and 33,142 and 3 in the V3V4 run, respectively (boxes not shown). Boxes include 25–75 percentile; whiskers include 5–95 percentile. Outliers not shown. + indicates the mean value; the line indicates the median value.

In total 64 milk extracts, 16 extraction controls (PCR‐grade water extracts), and two sequencing controls (MOCK standard and NTC) were sequenced in a V1V2 and a V3V4 run. First, clean reads of the sequenced milk samples from the different DNA extraction protocols were compared (Figure 1). The DNA yield and purity (*A*_280/260_) as well as the ratio *A*_260/230_ of the four extracted milk samples were measured and are listed in Table A1. The DNA yield and purity varied with each extraction method but also within the extraction method and triplicates. Particularly large variations were noted within the same sample and extraction day for P1 (DNeasy PowerSoil kit), P2 (DNeasy PowerFood kit), and P5 (modified QIAamp DNA Mini kit). The highest DNA concentrations were recorded using P3, the lowest concentrations were noted for P1. The median values of purity (A280/260) also showed considerable variability (Table A1).

Sequencing with primer pair V1V2 produced lower numbers of clean reads in all DNA extraction protocols, whereas primer pair V3V4 generally produced higher numbers of clean reads. The lowest median values were generated by P5 (modified QIAamp DNA Mini kit), resulting in 257 clean reads for V1V2 and 308 for V3V4. With a median of 268 clean reads for V1V2 and 983 for V3V4, P2 (DNeasy PowerFood kit) also generated a low read depth. The highest number of clean reads was generated by P6 (modified DNeasy Blood & Tissue kit), with a median of 6175 for the V1V2 run and 11,564 for the V3V4 run, respectively. P3 (QIAamp DNA Mini kit) achieved a median of 1616 clean reads in the V1V2 run and a median of 3482 clean reads in the V3V4 run. P4 (modified DNeasy Blood & Tissue kit) generated clean‐read results with median values of 1002 (V1V2) and 2527 (V3V4) in the pretrial. Finally, a median value above 1000 reads was set as the final criterion to select protocols for the main trial.

### 3.2 Main trial

#### 3.2.1 Quality control

**Figure 2:**
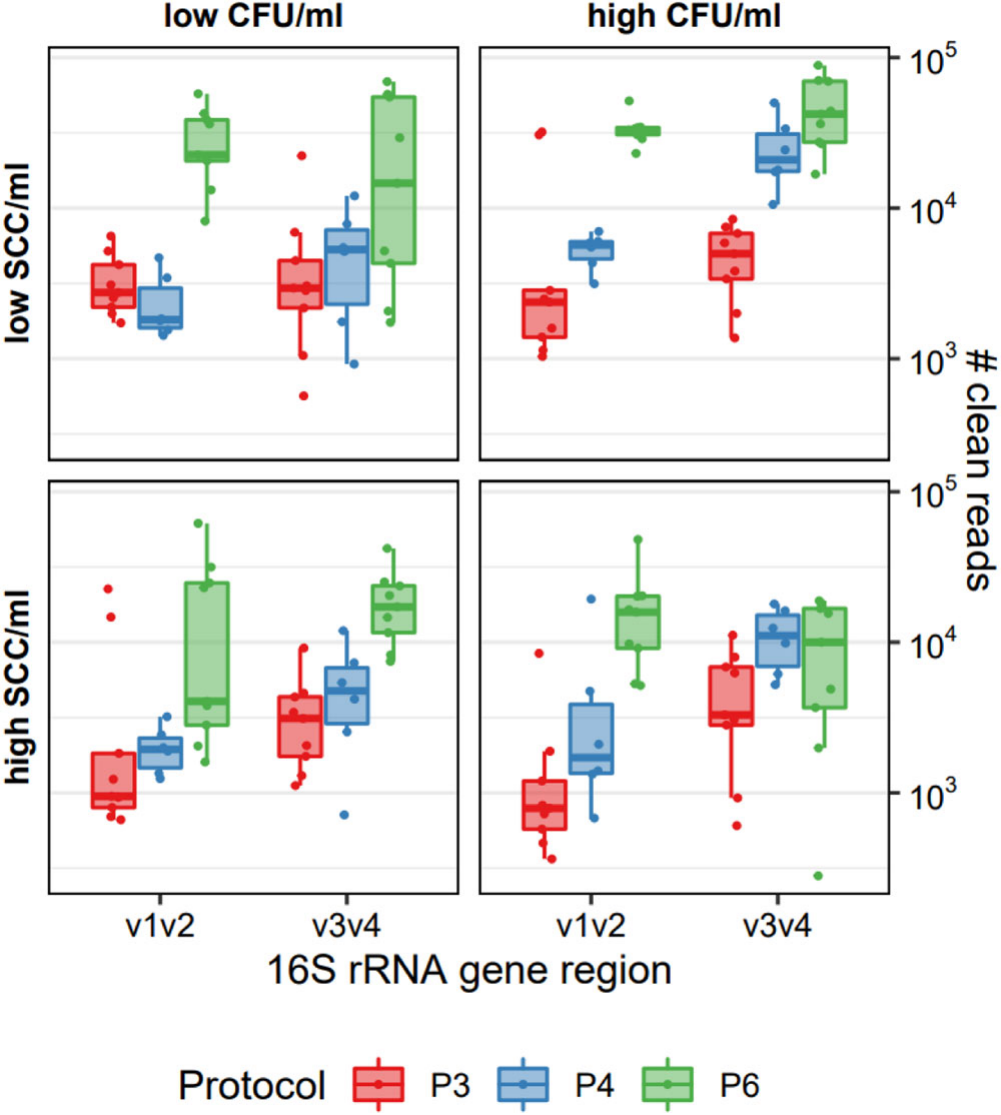
Clean reads of resequenced milk samples* with high/low cfu/SCC (V1V2 and V3V4) after applying different DNA extraction protocols (P3: QIAamp DNA Mini kit; P4, P6: modified DNeasy Blood & Tissue kit). All samples were extracted in two (P4) or three replicates (P3, P6) and sequenced in triplicates (1, 2, 3). *Prototype samples include unsuspicious milk (SCC−cfu−), and milk suspect of unspecific (SCC+cfu−; “high,” SCC+cfu+), or “latent” infection (SCC−cfu+). MOCK standard and NTC had clean reads of 29,222 and 0 in the V1V2 run and 23,300 and 224 in the V3V4 run, respectively (boxes not shown). cfu, Colony‐forming unit; NTC, no‐template control; rRNA, ribosomal RNA; SCC, somatic cell count.

Based on the median number of clean reads, P3, P4, and P6 were selected for further analysis. The milk extracts and PCR‐grade water extracts were resequenced with primer pairs V1V2 and V3V4 in triplicates. Read numbers of resequenced milk samples (*n* data sets = 96) separated by extraction protocol and milk prototype sample are shown in Figure 2. Also on repeat, the run with primer pair V1V2 produced lower numbers of clean reads than primer pair V3V4. Again, the highest numbers of clean reads were generated by P6 with a median of 22,810 for the V1V2 run and 17,643 for the V3V4 run, respectively. P4 achieved clean‐read results of 2263 (V1V2) and 8860 (V3V4), respectively. The lowest read depth was generated by P3 with a median of 1779 clean reads for V1V2 and 3341 for V3V4.

#### 3.2.2 Microbial composition

**Figure 3:**
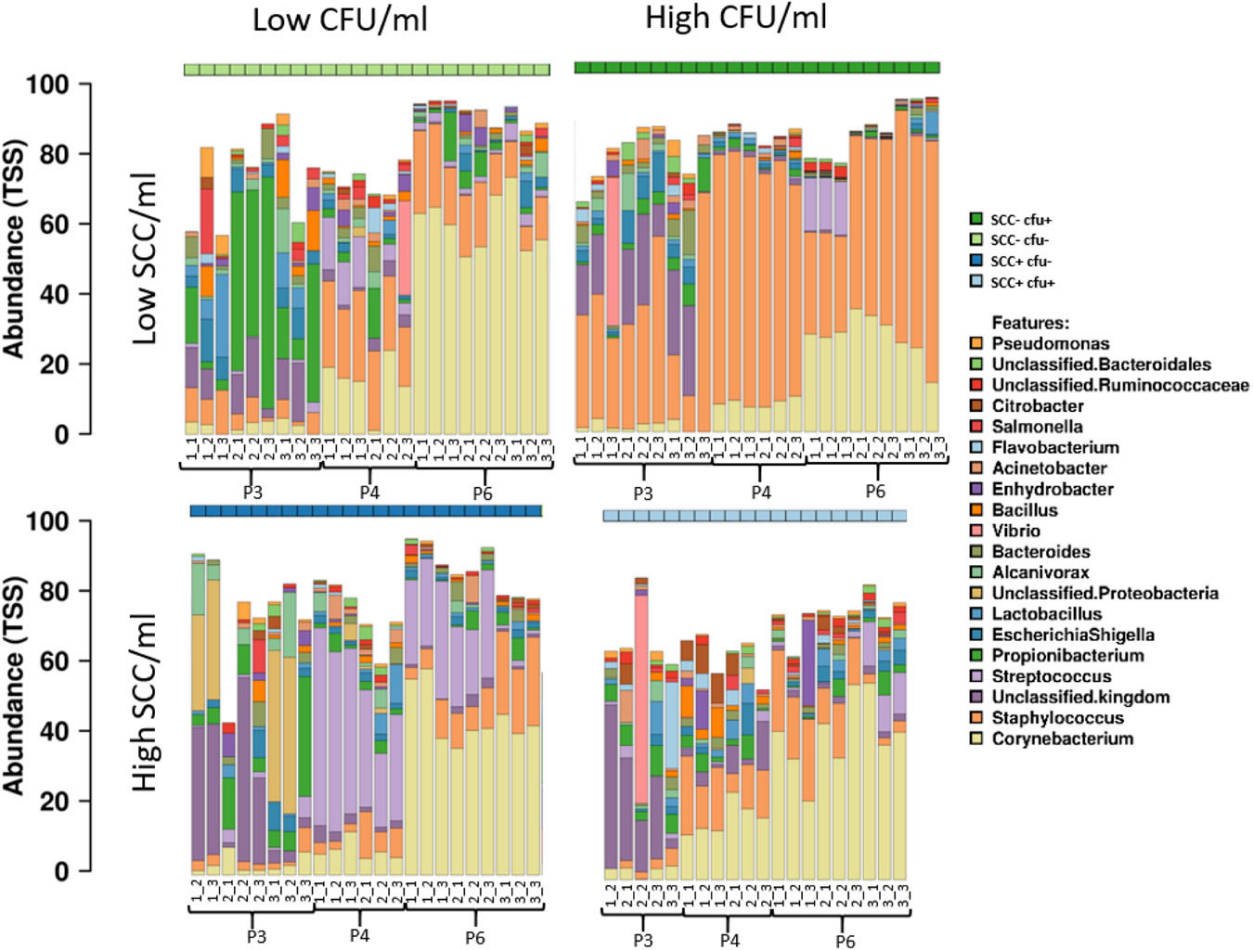
Microbial composition* in the V1V2 run of the four extracted milk samples^#^ with three different DNA extraction kits (P3: QIAamp DNA Mini kit; P4, P6: modified DNeasy Blood & Tissue kit). *The 20 most abundant taxa at the genus level (relative abundancy). Separate bar charts represent the result of triplicate extraction (duplicate in P4) and triplicate sequencing. Due to the remaining/unknown taxa bars do not sum to 100%. ^#^Prototype samples include unsuspicious milk (SCC−cfu−), milk suspect of unspecific (SCC+cfu−; SCC+cfu+), or “latent” infection (SCC−cfu+). The numbers below the bars indicate the extraction triplicate for each protocol within the milk sample (first number) and the respective sequencing triplicate (second number). cfu, Colony‐forming unit, SCC, somatic cell count; TSS, total sum scaling.

The distribution pattern of the bacterial genera in the resequenced milk samples extracted with different protocols is shown in Figures A4 and A5. The microbial composition of the four different prototype milk samples generated from both sequencing runs is shown in Figures 3 (V1V2) and 4 (V3V4). The 20 most relatively abundant taxa at the genus level are shown.

**Figure 4:**
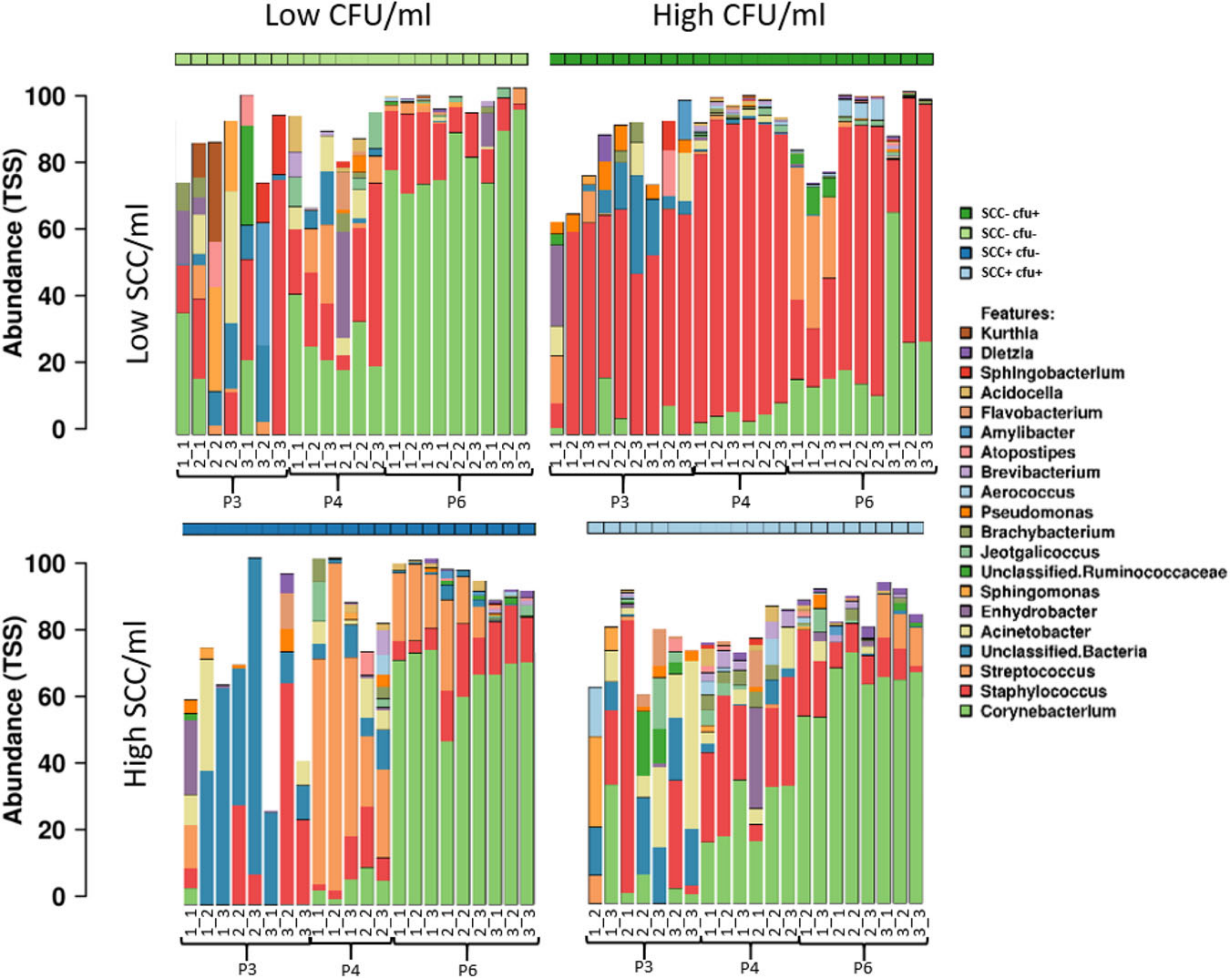
Microbial composition* in the V3V4 run of the four extracted milk samples^#^ with three different DNA extraction kits (P3: QIAamp DNA Mini kit; P4, P6: modified DNeasy Blood & Tissue kit). *The 20 most abundant taxa at the genus level (relative abundancy). Separate bar charts represent the result of triplicate extraction (duplicate in P4) and triplicate sequencing. Due to the remaining/unknown taxa bars do not sum to 100%. ^#^Prototype samples include unsuspicious milk (SCC−cfu−), milk suspect of unspecific (SCC+cfu−; SCC+cfu+), or “latent” infection (SCC−cfu+). The numbers below the bars indicate the extraction triplicate for each protocol within the milk sample (first number) and the respective sequencing triplicate (second number). cfu, Colony‐forming unit; SCC, somatic cell count; TSS, total sum scaling.

With regard to microbial composition, all protocols agreed in the fact that sample SCC−cfu− had the highest proportion of *Staphylococcus*, compared to the other samples. P4 and P6 agreed that sample SCC+cfu− has the highest proportion of *Streptococcus*. All protocols detected *Corynebacterium* spp. in all samples. However, the relative abundance of this genus differed strikingly: while *Corynebacterium* spp. was the most abundant genus with P4 in one (V1V2) to two (V3V4) samples and with P6 in three samples (V1V2, V3V4), this genus had only low relative abundance in P3. In addition, the percentage of the potential mastitis pathogens *Streptococcus* and *Staphylococcus* was lowest when the QIAamp DNA Mini Kit was used; this was observed in all four milk samples at V1V2 and in SCC−cfu− and SCC−cfu+ at V3V4. In P6, the high proportion of *Corynebacterium* was particularly evident. Furthermore, the highest proportion of unclassified ASVs was observed when using P3 (QIAamp DNA Mini kit). The proportion of unclassified or unknown ASVs was lower in P6 compared with P3 and P4. Within the 20 most abundant taxa, 8 were identical in the V1V2 (Figure 3) and the V3V4 run (Figure 4), including *Staphylococcus*, *Streptococcus*, and *Corynebacterium*, among others. Another nine genera, including several Proteobacteria, and also *Bacteroides*, *Bacillus*, *Lactobacillus*, and *Vibrio*, were abundant only in the V1V2 run, while V3V4 revealed 10 other genera like *Sphingomonas*, *Brevibacterium*, *Jeotgalicoccus*, *Aerococcus*, and others (Table A3).

#### 3.2.3 α‐ and β‐diversity

**Figure 5:**
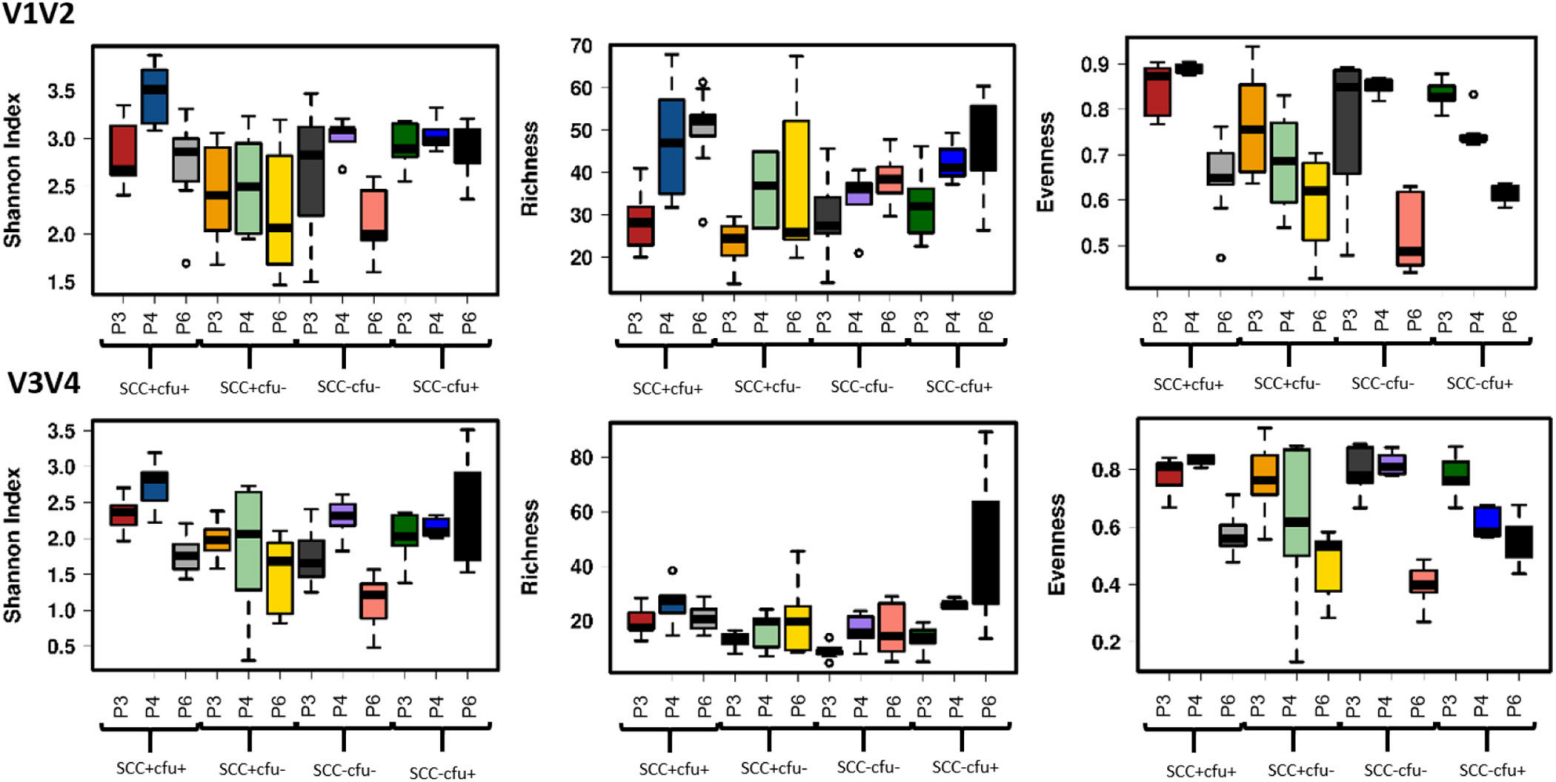
Comparison of microbial α‐diversity* based on ASV level (Shannon index, richness, evenness) of milk samples^#^ using DNA extraction kits (P3: QIAamp DNA Mini kit; P4, P6: modified DNeasy Blood & Tissue kit). *Comparison of runs with primer pairs V1V2 and V3V4. All samples were extracted in two (P4) or three replicates (P3, P6) and sequenced in triplicates. ^#^​​​​​​Prototype samples include unsuspicious milk (SCC−cfu−), milk suspect of unspecific (SCC+cfu−; SCC+cfu+), or “latent” infection (SCC−cfu+). ASV, amplicon sequence variant; cfu, colony‐forming unit; SCC, somatic cell count.

Figure 5 shows the microbial α‐diversity as revealed with both primer pairs on ASV level (Shannon index, richness, evenness). The figure includes the results of the resequenced milk extracts generated with the three DNA extraction protocols P3, P4, and P6. To account for protocol‐related differences in sequencing depth, samples were rarefied to protocol‐specific read depths. In the V1V2 run, this was a read depth of 302 (P3), 468 (P4), and 1302 (P6). In the V3V4 run, samples were rarefied to a read depth of 427 (P3), 612 (P4), and 1469 (P6). The highest diversity (Shannon index) was recorded for P4 (modified DNeasy Blood & Tissue kit) with milk sample SCC+cfu+ in the V1V2 as well as the V3V4 run. In the V1V2 run, Shannon indices were quite comparable between protocols, with median values between 2.4 and 2.8 for P3, between 2.5 and 3.5 for P4, and between 2.0 and 2.8 for P6. All protocols identified significant differences in Shannon indices between samples (*p* < 0.05), except P3 (*p* = 0.21). With P3, only V3V4 showed statistically significantly different results between samples, but only for diversity and richness, not for Shannon index (Table A4). In V1V2, P3 (QIAamp DNA Mini kit) revealed the lowest richness. In terms of evenness, the present ASVs seemed to be more evenly distributed after having been extracted with P3 and P4. Comparing V1V2 with V3V4, it is noticeable that the run with V1V2 produced higher Shannon diversity indices and a higher richness (up to >100 vs. up to 40 in P6). In the V3V4 run Shannon indices were slightly different between protocols, with median values between 1.6 and 2.3 for P3, between 2.0 and 2.8 for P4, and between 1.2 and 2.0 for P6.

With regard to the reproducibility of extraction and sequencing, the results were inconsistent. In V1V2 analysis, richness and Shannon indices were well reproducible (narrowly distributed) in replicates of samples SCC−cfu− and SCC−cfu+ for P4, and evenness indices were very well reproducible as well, except sample SCC+cfu−. For P6, richness was also well reproducible in replicates of samples SCC+cfu+ and SCC−cfu−. Reproducibility was generally low for P3, apart from evenness in SCC−cfu+. Compared to V1V2 analysis, samples were better or equally reproducible (narrowly distributed) in the V3V4 analysis with all protocols, except for sample SCC+cfu− (P3, P6) and sample SCC−cfu+ (P3, P4).

**Figure 6:**
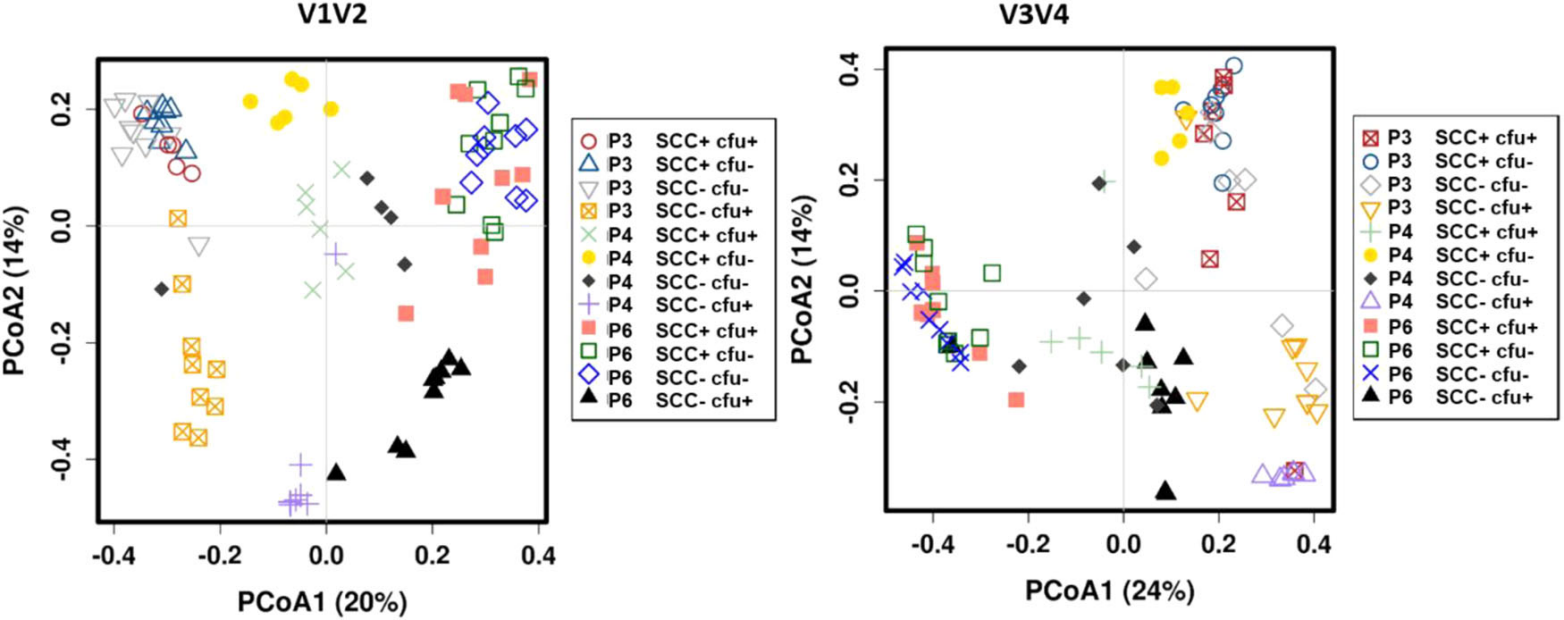
PCoA* of V1V2 and V3V4 amplicons representing the bacterial community of four milk samples^#^ extracted with three DNA extraction kits (P3: QIAamp DNA Mini kit; P4, P6: modified DNeasy Blood & Tissue kit). *Based on Bray–Curtis distance and ASV level. All samples were extracted in two (P4) or three replicates (P3, P6) and sequenced in triplicates. ^#^Prototype samples include unsuspicious milk (SCC−cfu−), milk suspect of unspecific (SCC+cfu−; SCC+cfu+), or “latent” infection (SCC−cfu+). ASV, amplicon sequence variant; cfu, colony‐forming unit; PCoA, principal coordinate analysis; SCC, somatic cell count.

To depict β‐diversity, we aimed to visualize in a PCoA the similarity between sample replicates when applying different protocols (Figure 6).

In V1V2, several replicates of sample–protocol combinations formed distinguishable point clouds, especially with P4, where sample SCC+cfu+, SCC+cfu−, and, with one outlier each, also sample SCC−cfu− and SCC−cfu+ were well separated. With P3 and P6, only sample SCC−cfu+ was separated from the others, while all other samples were clustered by protocol and not by sample type.

**Figure 7:**
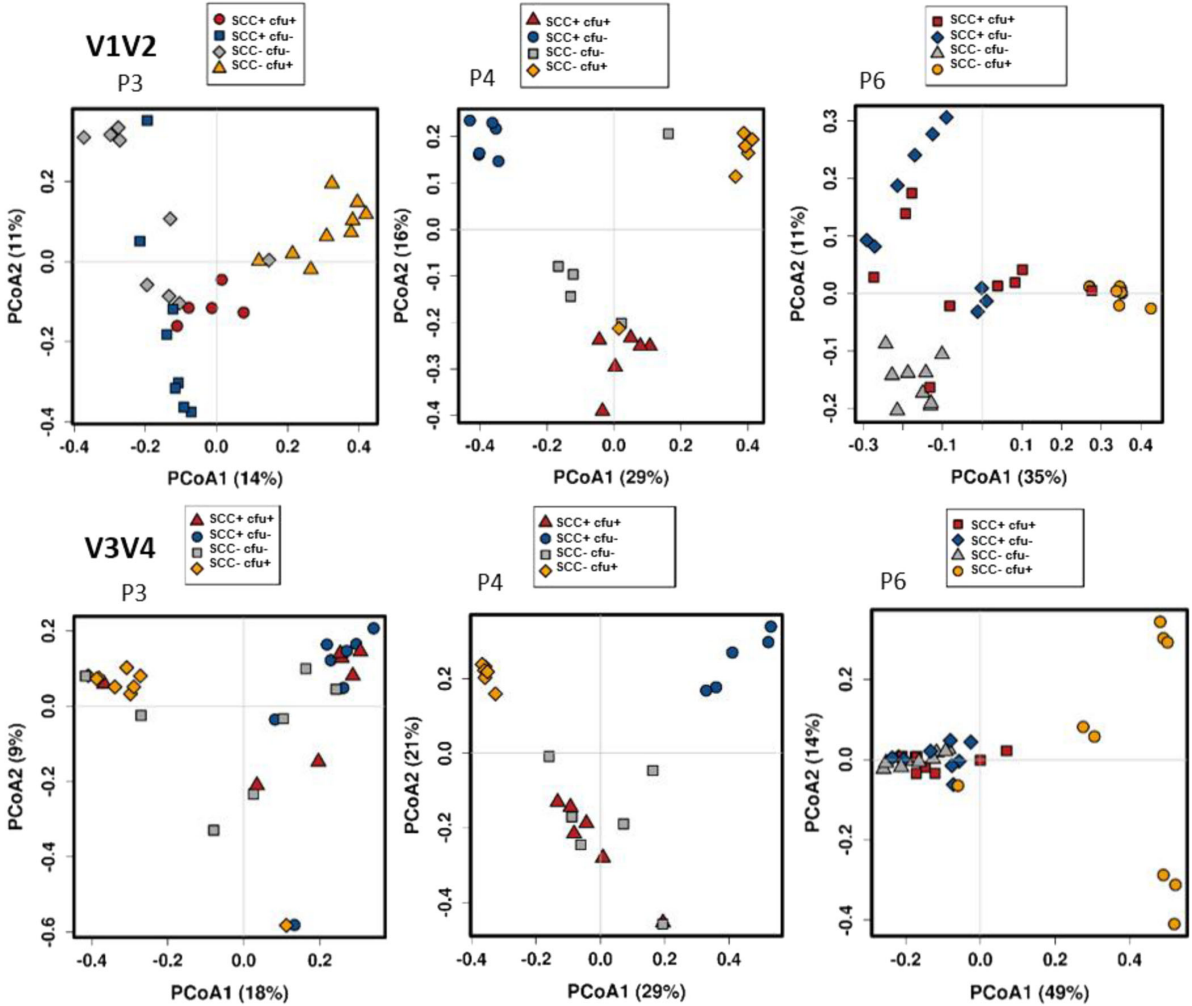
Kit‐based PCoA* of V1V2 and V3V4 amplicons representing the bacterial community of four milk samples^#^ extracted with three DNA extraction kits (P3: QIAamp DNA Mini kit; P4, P6: modified DNeasy Blood & Tissue kit). *Based on Bray–Curtis distance and ASV level. All samples were extracted in two (P4) or three replicates (P3, P6) and sequenced in triplicates. ^#^Prototype samples include unsuspicious milk (SCC−cfu−), milk suspect of unspecific (SCC+cfu−; SCC+cfu+), or “latent” infection (SCC−cfu+). ASV, amplicon sequence variant; cfu, colony‐forming unit; PCoA, principal coordinate analysis; SCC, somatic cell count.

For practical application, one would not mix sequencing results generated with different extraction protocols. Thus, to evaluate a successful separation of the samples types, six separated PCoAs were performed (Figure 7). Again, P4 showed the best separation of the milk samples. The reproducibility of the triplicates was highest (low level of dissimilarity) in sample SCC−cfu+, except for P6 at V3V4, where particularly the extraction triplicates differed very clearly from each other. With P3 and P4, the highest level of dissimilarity was shown for sample SCC−cfu−.

## 4 DISCUSSION

### 4.1 Main finding and general limitation

Our study revealed that different protocols differed substantially in their suitability to provide bacterial DNA for amplicon sequencing from the milk of the healthy and diseased udder gland, particularly in challenging samples with high SCCs. We aimed to validate different protocols under real conditions, not in artificially contaminated samples. However, these real conditions suffer from the fact that there is no “true” value for species composition since the community, as revealed by sequence analysis, might also include nonculturable bacteria and is thus not represented by results of microbial culture. Thus, we decided to choose prototype samples in a 2 × 2 design of main features (SCC, cfu; high vs. low) and to validate them in replicates against themselves, as described by Wen et al. (2017). To do so, we chose read numbers as the main unit to compare protocols (for reasons, see next section). This implies that we cannot separate between variability introduced by extraction or by sequencing. For that reason, we included resequencing of the same extracts to gain an idea of the variability introduced by sequencing. We emphasize that our findings on the variability of sequencing specifically refer to the matrix “milk extract.”

### 4.2 Selection of DNA extraction protocols

We selected published protocols that differed in terms of cell lysis (bead beating in P1, P2; chemical in the others), purification steps, and amount of initial milk volume (from 200 µl in P3 to 10 ml in P4, P6). Based on mean read numbers above 1000, P3, P4, and P6 were selected for resequencing. DNA yield and purity could not be used as a selection criterion. First, DNA yields from milk depict mainly mammalian DNA. Second, the NanoDrop instrument did not provide reliable measurements for all kits (Table A1). Overall, the DNA yield appeared to be very low, often below the quantification limit of 2 ng/µl. The low values might be due to interference with reaction kit components. However comparing the DNA yield of DNeasy PowerSoil kit and DNeasy PowerFood kit with those of Lima et al. (2018), where similar protocols were applied, physiological milk samples in that study recorded DNA concentration values of 11.5 and 16.8 ng/µl, respectively, while our unsuspicious milk sample SCC−cfu− had a maximum yield value of 1.37 (DNeasy Power Soil kit) and 3.17 ng/µl (DNeasy PowerFood kit). The extracted DNA from the four milk samples was tested for yield and purity. Overall, the DNA yield was very low, except for the yield values of P3 (QIAamp DNA Mini kit) with a maximum value of 18.44 ng/µl (median of three replicates). Nearly no extraction protocol provided recommended purity values in the range of 1.8–2.0 (Pirondini et al., 2010). Despite very low *A*_260/230_ purity values of extracts from all protocols, amplicon sequencing could be run successfully in most cases. Results were reproducible also with P4, despite a huge difference in *A*_260/230_ purity values between the replicates of sample SCC+cfu−. This hints toward a subordinate role of *A*_260/230_ quotients when judging the suitability of DNA extracts from milk for subsequent amplicon sequencing, at least when using the protocols used in this study.

### 4.3 Choice of primer pairs

**Figure A2:**
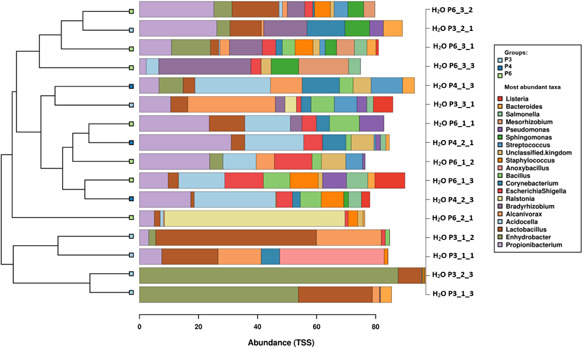
Microbial composition* and clustering of the resequenced water extracts (*n* = 16) after filtering using the different DNA extraction protocols (P3: QIAamp DNA Mini kit; P4, P6: modified DNeasy Blood and Tissue kit) in the sequencing run with primer pair V1V2. *The 20 most abundant taxa at the genus level are shown. All water samples were run as controls during the milk sample extractions and were extracted in two (P4) or three replicates (P3, P6) and sequenced in triplicates. The first number following the P labels the extraction replicate, the latter number labels the sequencing replicate. TSS, total sum scaling.

**Figure A3:**
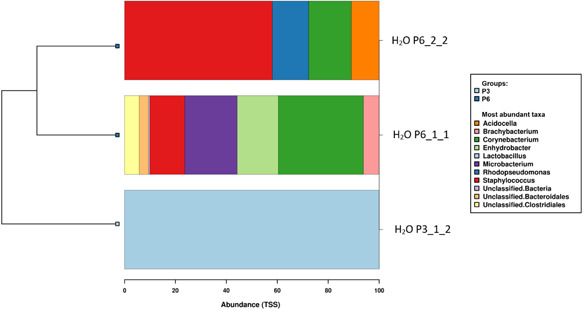
Microbial composition* and clustering of the resequenced water extracts (*n* = 3) after filtering using the different DNA extraction protocols (P3: QIAamp DNA Mini kit; P6: modified DNeasy Blood and Tissue kit) in the sequencing run with primer pair V3V4. *The 20 most abundant taxa at the genus level are shown. All water samples were run as controls during the milk sample extractions and were extracted in two (P4) or three replicates (P3, P6) and sequenced in triplicates. The first number following the P labels the extraction replicate, the latter number labels the sequencing replicate. TSS, total sum scaling.

The 16S rRNA gene was amplified using two primer pairs for the hypervariable regions V1V2 and V3V4. The primer we used for V1V2 was already shown to be suitable for sequencing of milk samples by Oikonomou et al. (2012), while Cremonesi et al. (2018) targeted the V3V4 region, but used other primers. In our study, across DNA extraction protocols, V1V2 generated fewer reads than V3V4, but overall higher diversity measures (Shannon Index) were obtained. However, a larger number of water extracts (*n* = 16) presented with more than 500 sequence reads in the V1V2 run than in the V3V4 run (*n* = 3) (Figures A2 and A3), indicating that V1V2 is more susceptible to contamination.

### 4.4 Plausibility check and data curation

In general, the detected ASVs in the V1V2 and V3V4 run in our study were similar to the findings of Lima et al. (2018). As recommended by Wen et al. (2017), some ASVs in the V1V2 and V3V4 sequencing run (here: single hits and those that only occurred in the water but not in the milk extracts) were manually removed (for details see Appendix A). Possible explanations for the high number of unreproducible ASVs are sequencing errors, artifacts, or index swapping among/between cosequenced samples (Bakker et al., 2012; Costello et al., 2018; Kunin et al., 2010), the latter leading to a sequence, which is erroneously attributed to the wrong barcode. Index swapping became a particular focus since ASVs were generated that are not typically found in milk but partly fit other specimens sequenced in the same run. Thus, we run a plausibility analysis, which verified whether certain taxa are generally present in bovine milk or result more likely from cosequenced samples. In the V1V2 run, soil and human skin samples, and in the V3V4 run, extracts from sponges from the ocean and human periodontitis samples were sequenced. ASVs of the genus *Aquabacterium* and *Pelomonas* were found in the V3V4 run, which is usually abundant in water (Gomila et al., 2007; Hirose et al., 2020), so this could be due to an index swap with the sponges from the ocean. These ASVs were removed to avoid overestimation of the abundance of genera, as already reported by Gomez‐Alvarez et al. (2009). In the V1V2 run, ASVs of the genus *Acidocella* were removed as they are not attributed to milk but rather to soil samples (Dore et al., 2003), suggesting an index swap with cosequenced soil samples. However, the presence of *Acidocella* also in the V3V4 run does not allow to draw conclusions. Here, *Acidocella* was not excluded, since it occurred in less than three water samples. Thus, either our exclusion criteria were too lax, including an environmental contaminant in the V3V4 data, or they were too sharp, excluding an abundant milk genus from V1V2 data. Both views might be supported by Lopez Leyva et al. (2021), where *Acidocella*, on the one hand, was found among the most abundant genera from human breast milk in one study, whereas, on the other hand, it was not reported as an abundant genus in 18 other studies. *Acidocella* was consistently found with both primer sets after extraction with all investigated protocols. The same applies to *Alcanivorax* in V1V2: one ASV of *Alcanivorax* was found in more than two water extracts (and therefore excluded), while other ASVs of *Alcanivorax* gave the impression that *Alcanivorax* was one of the 20 most abundant genera in milk in the V1V2 run after extraction with all investigated protocols.

In sequencing runs consisting of milk extracts only, index swaps might also take place, but would not be as noticeable because no untypical genera would be present. Thus, our report allows us to get an impression of the frequency of this known source of error in genera abundance. With 1.64% of reads for V1V2 and 1.1% of reads for V3V4, respectively, only a small number of reads was probably affected by index swap. The numerous ASV detections in the PCR‐grade water extracts, which were run as controls, may also be due to index swaps and artifacts, and also due to contamination during DNA extraction (partly already introduced by kit reagents) (Salter et al., 2014) and sequencing (Tanner et al., 1998). There was a lack of reproducibility within water extractions; when ASVs were present, they were usually present in the same kit but not necessarily in extraction replicates of the same sample, indicating that contamination occurred rather by kit ingredients than by handling. In addition, it is striking that the genus *Bradyrhizobium* was predominant in only one extraction kit in the V1V2 run (P3, QIAamp DNA Mini kit), and the genera *Ralstonia* and *Caulobacter* were only present in the modified DNeasy Blood & Tissue kit (P6) in the V3V4 run. These genera have previously been reported as contaminating genera in negative “blank” controls (Salter et al., 2014). Both water data sets (V1V2, V3V4) were primarily dominated by *Propionibacterium acnes*, possibly due to an index swap with cosequenced samples from human skin (V1V2) and oral cavity (V3V4), as *P. acnes* is a commensal colonizer on the skin and in the oral cavity (Perry & Lambert, 2011). However, as a skin commensal, this bacterium might have also entered the samples by laboratory personnel. Altogether, our criteria led to its removal.

### 4.5 Number of clean reads and accordance with colony counts

Milk extracts had partially low read numbers in both runs. This may be due to the challenging feature of the medium milk in the previous amplification since fat, protein, and calcium molecules, as well as bacterial and mammalian debris, may interfere (Powell et al., 1994; Schrader et al., 2012; Wilson, 1997). Interestingly, in the V1V2 run of the pretrial, P1 and P2 did not perform very well in terms of clean read number, although these bead beating methods had already been proven suitable in previous studies such as that of Lima et al. (2018). However, Lima et al. (2018) added an intensive wash step to the DNeasy PowerSoil kit standard protocol, which might explain the more favorable results, compared to our study. Despite this adaption, the authors also pointed to the fact that DNA extraction in milk from healthy udders still has to be improved.

Consideration should also be given to the amount of milk used at the beginning of DNA extraction, which varied among DNA extraction protocols (from 200 µl to 10 ml initial volume). The difference in initial volume can lead to losses in the total amount of extractable bacteria, especially at low input volumes (Zhang et al., 2018).

Cultural bacterial counts might or might not agree with read numbers. With P4, the samples with the fewest cultural cfu also had the fewest sequence reads. By contrast, low SCCs—indicating a healthy, uninfected udder—did not result in a lower number of bacterial reads: SCC−cfu+ had significantly more bacterial reads than sample SCC+cfu+. At first glance, this might appear rather counterintuitive, since high SCCs mostly indicate bacterial infection, raising the expectation of high bacterial read numbers. However, the somatic cells in the quarter with high SCCs might have impaired the extraction process by providing mammal DNA that competed at the DNA binding column. High numbers of bacterial reads in milk from healthy udders are not implausible, since milk is not expected to be sterile, as also reported by Metzger et al. (2018).

In P3 and P6, there was no association between cultural cfu and sequence read number. Instead, the read number was low when the SCC was high, regardless of whether there were many or few culturable bacteria in the sample. Only within the samples with low SCC, an association was seen at P6 (more cfu, more reads), while in SCC‐rich samples this association was even inverse and samples with less cultural cfu recorded more sequence reads, especially when using V3V4 primers.

### 4.6 Comparing selected extraction protocols (P3, P4, P6) based on microbial composition

Suitable protocols should reveal the same, plausible microbial composition for the same sample, at least after data curation (see respective section). All DNA extraction protocols agreed on the fact that sample SCC−cfu+ was dominated by the genus *Staphylococcus*. This is consistent with the classification of coagulase‐negative staphylococci, such as *Staphylococcus epidermidis* and *Staphylococcus chromogenes* as minor pathogens in mastitis, lacking a substantial increase of SCCs (Taponen & Pyörälä, 2009). In sample SCC+cfu−, P4 and P6 detected mainly reads of the genus *Streptococcus* (*St*.). This finding supports the view of certain *Streptococcus* spp. as major pathogens (e.g., *St. uberis*) that might cause disease at low infection dose already (Nickerson et al., 1990). Unfortunately, species information from amplicon sequencing is limited because identification at the species level was not possible in all cases. However, in 17 out of 24 replicates of sample SCC+cfu−, ASVs of the major pathogen *St. uberis* were detected. P3 was not able to provide that information: most of the ASVs in that sample belonged to unclassified bacteria when using V3V4 primers, and were attributed to unclassified Bacteroidales and Proteobacteria with V1V2 primers.

In sample SCC+cfu+, no obvious major pathogen was identified, although cfu and SCC were high. This is in accordance with the result of the cultural investigation (mixed culture) and could indicate a multifactorial inflammatory process, facilitating unspecific bacterial infection in the udder of a predisposed cow. A large proportion of generated ASVs (P4 and P6) were mainly of the genus *Corynebacterium*. The cultural detection of this genus in cows of the observed herd was associated with significantly increased SCC, pointing toward a potentially virulent clone. However, sample SCC−cfu− had a comparably high relative abundance of *Corynebacterium*, but lacked inflammatory reaction, which might underline the role of individual disposition. To mention a side note, sample SCC−cfu+ had a much lower abundance of *Corynebacterium* compared to sample SCC−cfu−, which was taken from a quarter of the same cow. This demonstrates once more that bacterial composition is specific for quarters, not for cows, which is consistent with the anatomical separation of udder quarters.

Important minor or major mastitis pathogens like *Staphylococcus*, *Streptococcus*, and *Corynebacterium* were found with both primer sets, as also described by Falentin et al. (2016) and Kuehn et al. (2013). Several genera of the phylum Proteobacteria and also genera, such as *Bacillus* and *Lactobacillus*, were exclusively detected in the V1V2 analysis. This is in agreement with Oikonomou et al. (2014). Interestingly DNA of strict anaerobes like *Bacteroides* was also present in the milk samples, which was also reported by Oikonomou et al. (2014). The detection of *Salmonella* spp. might be surprising at first glance but is consistent with an outbreak on the farm at the time of sampling (confirmed by the National Reference Laboratory, personal information). In agreement with Yeung et al. (2011), V3V4 tended to detect more environmentally associated bacteria, including also *Aerococcus* and *Brevibacterium*, which are known environmental mastitis pathogens (Lima et al., 2018; Sun et al., 2017).

### 4.7 Comparing extraction protocols based on α‐ and β‐diversity

To reliably compare diversity values, rarefication is an important step. Here, we decided to rarefy within, not across protocols, since we assume that read depth contributes to the suitability of a protocol. Thus, rarefying the read number of all protocols according to the protocol with the lowest read number would have artificially impaired the overall results of the other protocols. For P3, rarefied read numbers of 302 (V1V2) and 427 (V3V4), respectively, raise doubts on its suitability to provide representative results whenever challenging samples are compared. Despite the low read depth in P3, Shannon diversity index values showed comparable ranges in all three protocols. However, no separation between sample prototypes was achieved with P3. Evenness was higher (closer to 1) in P3 and P4, indicating a more even distribution, that is, less different ASVs. Similar evenness values in quarter milk samples were reported by Andrews et al. (2019), where especially in quarters of healthy cows the evenness was high. Only P3 and P4 confirmed the latter observation. Shannon diversity index values resembling those of our study (2.5–3.5) were described by Metzger et al. (2018a). Other studies reported higher values in the range of 3–7.5 (Ganda et al., 2016; Metzger et al., 2018b; Oikonomou et al., 2012).

Only P4 was able to separate four (V1V2) or three (V3V4) different sample conditions from each other in the PCoA. The insufficient separation in P3 could be due to the lack of pretreatment. However, the pretreatment, which was successfully combined with the DNeasy Blood & Tissue kit (P4) was also combined with P3 in the pretrial, but did result in a comparatively low number of reads and was therefore excluded (P5 in Figure 1). P6 included a pretreatment but also lacked the ability to separate the sample types in a PCoA. The protocol was originally optimized for *M. avium* subsp. *paratuberculosis*. Based on structural similarity between the cell wall of *Mycobacterium* spp. and *Corynebacterium* spp. (Poetsch et al., 2011), one might therefore suspect that P6 enriches or preferentially extracts *Corynebacterium* spp., which might increase the similarity between actually different samples. The assumption that one species was enriched is further confirmed by a low evenness value of the samples extracted with that protocol. Thus, modification steps from P4 affecting cell lysis (prolonged lysozyme incubation and boiling lysis) might simply separate more effectively between sample types than modifications affecting bacterial distribution in the cream and milk fractions (P6). For *Mycobacterium* spp., the most satisfactory results were obtained by combining both modifications (Mayer, 2018). Nevertheless, this would have further increased the processing effort and costs. The performance of the QIAamp DNA Mini kit (P3) might be also improvable by combining this kit with another pretreatment, as described by Goldschmidt et al. (2014). P4 generates comparatively high costs per reaction (23.10€ vs. 7.40€ for P3 and 6.45€ for P6). The processing time is approx. 225 min for P4 versus 60 min for P3 and 335 min for P6, with a hands‐on time of approx. 85 min for P4 versus 35 min for P3 and 50 min for P6, respectively. However, the much better results seem to justify costs and efforts.

### 4.8 Reproducibility of methods

**Figure A4:**
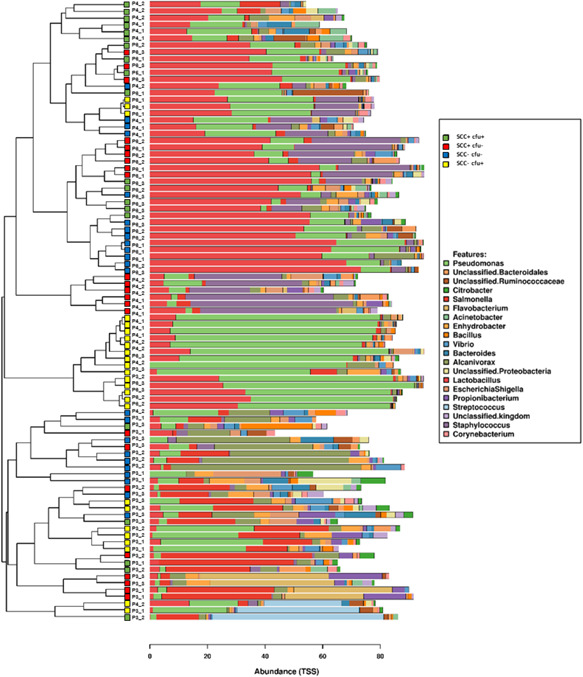
Microbial composition* and clustering of the four resequenced milk samples^#^ using the different DNA extraction protocols (P3: QIAamp DNA Mini kit; P4, P6: modified DNeasy Blood and Tissue kit) in the sequencing run with primer pair V1V2. *The 20 most abundant taxa at the genus level are shown. All samples (*n* = 91) were extracted in two (P4) or three replicates (P3, P6) and sequenced in triplicates. ^#^Prototype samples include unsuspicious milk (SCC−cfu−), milk suspect of unspecific (SCC+cfu−; SCC+cfu+), or “latent” infection (SCC−cfu+). The number following the protocol number labels the extraction replicate; the same symbols and numbers represent sequencing replicates. cfu, Colony‐forming unit; SCC, somatic cell count; TSS, total sum scaling.

**Figure A5:**
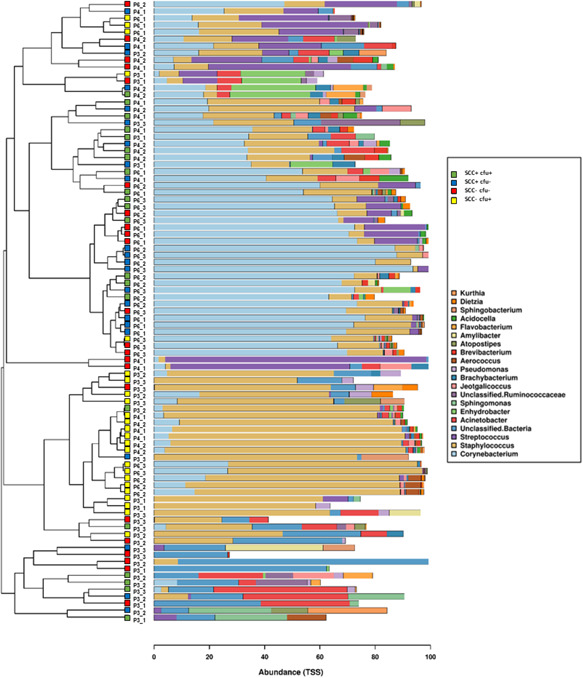
Microbial composition* and clustering of the four resequenced milk samples^#^ after filtering** using the different DNA extraction protocols (P3: QIAamp DNA Mini kit; P4, P6: modified DNeasy Blood and Tissue kit) in the sequencing run with primer pair V3V4. *The 20 most abundant taxa at the genus level are shown. All samples (*n* = 89) were extracted in two (P4) or three replicates (P3, P6) and sequenced in triplicates. ^#^Prototype samples include unsuspicious milk (SCC−cfu−),  milk suspect of unspecific (SCC+cfu−; SCC+cfu+), or “latent” infection (SCC−cfu+). The first number following the P labels the extraction replicate; the same symbols and numbers represent sequencing replicates. cfu, Colony‐forming unit; SCC, somatic cell count; TSS, total sum scaling.

In the following, the reproducibility of P3, P4, and P6 is discussed in terms of read numbers, α‐diversity indices, and PCoA results. As mentioned in the limitation section, there is no “true” value of read numbers or bacterial composition, and all extraction replicates combine extraction variability with sequencing variability. To overcome this problem, we provide comparative values of resequencing for the same extract. We refer to the results of resequencing as “sequencing variability” and the results of re‐extraction plus resequencing as “total variability.” With primer pair V1V2, maximum sequencing variability, measured as the maximum difference in clean‐read numbers, was equal to 98%–139% of the maximum total variability between extracts for P3, 62%–96% for P4, and 62%–75% for P6. With primer pair V3V4, maximum sequencing variability was equal to 71%–88% of the maximum total variability for P3, 44%–171% for P4, and 41%–89% for P6. Altogether, there were only 2 out of 24 cases where extraction seemed to introduce more variability than the sequencing step (sequencing accounted for less than 50% of total variability there). Thus, if resources are limited, we recommend to rather repeat sequencing instead of extraction. For the unmodified P3, extraction contributed insignificantly to the total variability seen between replicates sequenced with primer pair V1V2 (−27 to 1.8 percentage points, if maximum sequencing variability was set to 100%). In all other conditions, at best both steps (extraction plus sequencing) would be performed in duplicates. This gets obvious also from the fact that genus composition differs remarkedly between some of the replicates, as shown in Figures A4 and A5. With regard to the reproducibility of α‐diversity indices, P4 showed the lowest variability, that is, the most reproducible results. Within P4, sample SCC+cfu− with the most difficult conditions (high SCC, low cfu) showed the lowest reproducibility. With regard to β‐diversity, replicates of SCC−cfu+ showed remarkably large variation in P6.

Initially, we were afraid that modifications might decrease reproducibility since protocols other than manufacturer instructions are often suspected to lack standardization and thus repeatability (Hermans et al., 2018). With comparable results gained from extractions performed on two different days, we had no reason to doubt the reproducibility of our modifications. However, person‐to‐person‐variability was not assessed in our study.

## 5 CONCLUSION

We conclude that the different extraction protocols achieve various reproducible results in terms of α‐ and β‐diversity as well as read number, even on the replicate level of extraction and sequencing. With simple milk samples (SCC−cfu+) most protocols generated well reproducible results. Especially in cases of nonspecific mastitis (sample SCC+cfu+ and SCC+cfu−) more effort and costs have to be taken up to achieve reproducible results. In any case, we recommend performing a plausibility check (removing single hits and other unrepresentative ASVs), especially if samples of other specimens had been cosequenced as well. Furthermore, we recommend extracting and/or sequencing duplicates of such challenging samples. Notwithstanding, 16S rRNA gene sequencing is an important tool to advance our knowledge of culture‐independent bacteria in the bovine udder gland. Further research is needed to develop suitable DNA extraction kits or protocols to obtain reliable and reproducible results to cover the milk microbiome, taking into account applicability in terms of costs and efforts.

## AUTHOR CONTRIBUTIONS

**Julia A. Schwenker**: Conceptualization (supporting); data curation (equal); formal analysis (lead); visualization (lead); methodology (supporting); software (equal); investigation (lead); writing—original draft (lead). **Meike Friedrichsen**: Conceptualization (supporting); data curation (supporting); investigation (supporting); methodology (supporting); writing—review and editing (supporting). **Silvio Waschina**: Visualization (supporting); methodology (supporting); writing—review and editing (supporting). **Corinna Bang**: Methodology (supporting); investigation (supporting); data curation (equal); formal analysis (supporting); software (equal); writing—review and editing (supporting). **Andre Franke**: Data curation (supporting); methodology (supporting). **Ricarda Mayer**: Methodology (supporting); investigation (supporting). **Christina S. Hölzel**: Conceptualization (lead); formal analysis (supporting); funding acquisition (lead); methodology (lead); project administration (lead); software (supporting); supervision (lead); visualization (supporting); writing—original draft (supporting); writing—review and editing (lead).

## CONFLICTS OF INTEREST

None declared.

## ETHICS STATEMENT

None required.

## Figures and Tables

**Figure A1:**
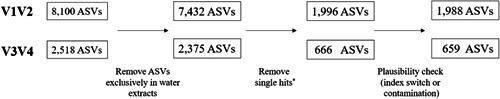
Flowchart of the removal of ASVs from both runs V1V2 and V3V4, which were subsequently used in Calypso for microbial analysis. *Only one ASV over the entire milk data set. ASV, amplicon sequence variants.

## Data Availability

All data are provided in full in this paper, except for raw data (clean reads and amplicon single variants), which are available in the Zenodo repository at https://doi.org/10.5281/zenodo.6340208.

## APPENDIX A

A total of 8100 ASVs were generated for V1V2 and 2518 ASVs for V3V4. ASVs of the resequenced extracts were removed from the abundance tables if they occurred exclusively in the water extracts and not in the extraction replicates of the milk extracts. A flow chart showing the different steps of which ASVs are removed can be seen in Figure A1. We expect that most of these ASVs would have also been removed by the subsequent filtering steps in Calypso, which exclude ASVs with less than 500 sequence reads and 0.01% relative abundance across all samples. However, we decided to remove these ASVs manually and to report their number, to make the process transparent. Thus, 668 ASVs were removed from the V1V2 run and 143 from the V3V4 run. Furthermore, ASVs were removed if they were found only as a single hit (one out of the triplicates in one single milk extract, which means one result over the entire milk data set). This affected 5436 and 1708 ASVs in V1V2 and V3V4, respectively. ASVs occurring in the milk extracts and more than two replicates of the water extracts were checked for plausibility (index switch or contamination). This applied to 22 ASVs (V1V2) and 14 ASVs (V3V4), respectively. ASVs of genera that are not reported to appear in milk were deleted. Accordingly, two ASVs of the genus *Acidocella* (together 9270 reads summed up across all water and milk replicates), as well as one ASV of the genus Alcanivorax (13,314 reads across replicates), were removed in the V1V2 abundance table. Two ASVs of the genus *Aquabacterium* (together 9969 reads across replicates) and one ASV of the genus *Pelomonas* (5856 reads across replicates) were removed in the V3V4 abundance table. Two ASVs of the genus *Escherichia/Shigella* and *Staphylococcus* were found in more than 33% of the water extractions. Therefore, these ASVs were considered as contaminants and were removed from the data set before further analysis was performed. With each primer pair, one ASV of *P. acnes* (recently renamed *Cutibacterium acnes*) was very frequently found in water extracts, more precisely in 22 of 24 extracts in V1V2 and 11 of 24 extracts in V3V4, reaching considerable read numbers (up to 2554) in the PCR‐grade water extracts. These two ASVs were also deleted. Two rarer ASVs of *P. acnes* did not occur in water, but occurred only occasionally (means: only in one of the extraction triplicates) in low read numbers (below 100 per milk sample) and were considered as other contaminants or sequencing errors of the frequent ASVs, thus being deleted as well. Finally, 1988 ASVs of the V1V2 run and 659 ASVs of the V3V4 run were used for further analysis. At the final filtering step in Calypso software, five samples at V1V2 were discarded since they had less than 500 reads. In the run with V3V4 seven samples were removed due to a low read number. After filtering in Calypso, the water extracts were examined for possible clustering between the extraction kits to detect possible kit‐specific contaminants. After excluding ASVs below 500 reads, the V1V2 and V3V4 water data set still contained 16 and 3 samples, respectively. However, most ASVs from water extracts were unsystematically scattered over diverse kits and repetitions. Only for P6 in extraction 1, the sequencing triplicates clustered together. However, at the same time, several of them clustered—more closely—with P4 (see supplementary data, Figures A2 and A3). The most dominant genera in these sequence sets were *Propionibacterium* and *Acidocella*. In general, the dominant genera in the V1V2 run of water extracts were *Enhydrobacter*, *Propionibacterium*, and *Lactobacillus*; the V3V4 run detected *Lactobacillus* as well as *Corynebacterium* and *Staphylococcus* in the water extracts (Figure A3).

**Table A1:** Yield^a^ and purity of extracted DNA of six extraction protocols (P1–P6, for description see Table 2) of four milk samples^b^ (values are given as median (min–max) of three measurements)

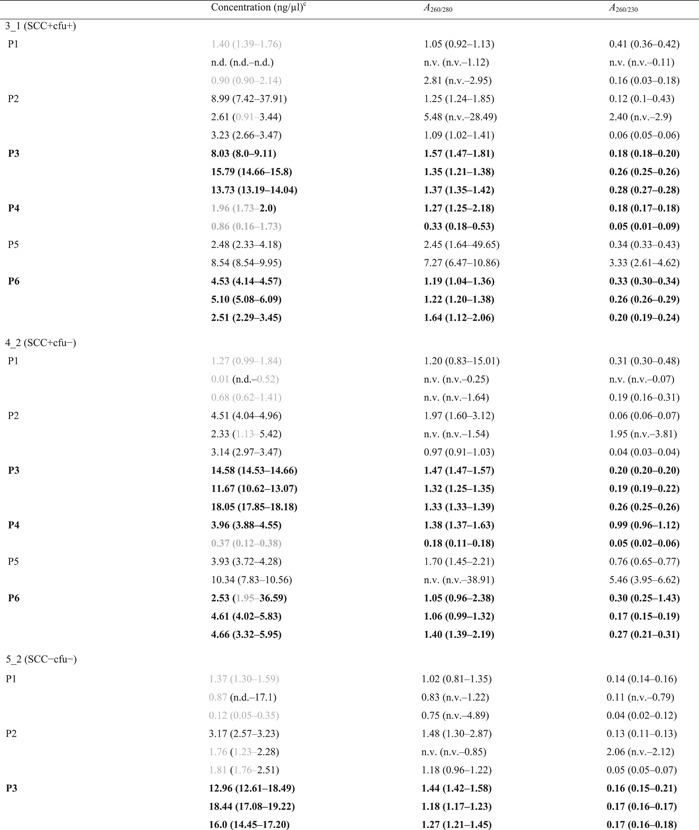

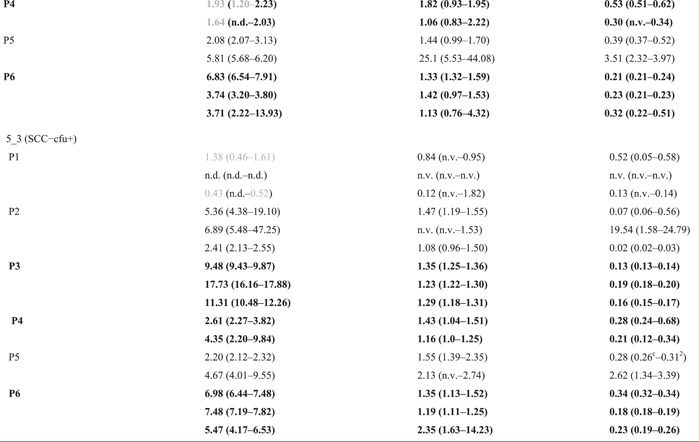

*Note*: The final elution volume was 200 µl, except P1 and P2 (100 µl). All samples were extracted in two (P4) or three replicates (P3, P6) and sequenced in triplicates. Gray font: below quantification limit of 2 ng/µl. Bold: protocols were chosen for the main trial. Control extracts of nuclease‐free water of the respective protocol varied between n.d. and a maximum value of 6.92 ng/ml (median value of 2.11 ng/ml).

Abbreviations: cfu, Colony‐forming unit; n.d., not detectable; n.v., no measurable value; SCC, somatic cell count.

^a^ Yield: median value of DNA per eluate (ng/µl).

^b^ Prototype samples include unsuspicious milk (SCC−cfu−; 5_2), milk suspect of unspecific (SCC+cfu−; 4_2; “high,” but mixed cfu+SCC+; 3_1), or “latent” infection (SCC−cfu+; 5_3).

^c^ DNA concentrations refer to the eluate and not to the original milk sample, and were therefore not corrected for different starting volumes.

**Table A2:** Quarter foremilk samples of 24 cows with data of somatic cell count (SCC/ml milk) and colony‐forming units (cfu/ml milk)

Cow no.	Sample name in this study	Quarter	cfu/ml^a^	SCC/ml
6634	1.1	LF	55	609,000
	1.2	RF	25	67,000
	1.3	RH	5	711,000
	1.4	LH	200	322,000
6505	2.1	LF	0	98,000
	2.2	RF	230	135,000
	2.3	RH	1300	267,000
	2.4	LH	120	112,000
6558	3.1^b^	LF	725	393,000
	3.2	RF	410	174,000
	3.3	RH	180	170,000
	3.4	LH	85	156,000
6770	4.1	LF	195	126,000
	4.2^b^	RF	90	318,000
	4.3	RH	4000	606,000
	4.4	LH	275	194,000
6564	5.1	LF	5	59,000
	5.2^b^	RF	65	86,000
	5.3^b^	RH	495	117,000
	5.4	LH	285	149,000
6860	6.1	LF	235	180,000
	6.2	RF	160	979,000
	6.3	RH	330	472,000
	6.4	LH	65	479,000

Abbreviations: cfu, Colony‐forming unit; LF, left front quarter; LH, left hindquarter; RF, right front quarter; RH, right hindquarter; SCC, somatic cell count.

^a^ Mean value of colony‐forming units (cfu/ml milk) of incubated blood agar plates (1:10 diluted and undiluted).

^b^ Selected quarter milk samples.

**Table A3:** Consensus between extraction protocols for the 20 most abundant taxa after sequencing with primer pairs V1V2 and V3V4^a^

V1V2 plus V3V4	Only V1V2	Only V3V4
***Acinetobacter***	***Alcanivorax***	*Acidocella* b, c
***Corynebacterium***	***Bacillus***	*Aerococcus* d
***Enhydrobacter***	***Bacteroides***	*Amylibacter* e
Ruminococcaceae^f^ (Family)	***Citrobacter***	*Atopostipes* d
*Flavobacterium* g	***Escherichia/Shigella***	*Brachybacterium* h
*Pseudomonas* i	***Lactobacillus***	*Brevibacterium* d
***Staphylococcus***	***Propionibacterium***	*Dietzia* j
***Streptococcus***	*Salmonell*a^k^	*Jeotgalicoccus* d
	*Vibrio* l	*Sphingobacterium* j
		*Sphingomonas* d

*Note*: Bold represents consensus in all sample prototypes and with all protocols.

Abbreviations: cfu, Colony‐forming unit; SCC, somatic cell count.

^a^ All taxa were found in all sample prototypes, but not with all protocols and primer sets.

^b^ Consensus in V3V4, except for sample SCC−cfu+.

^c^ This genus was deleted from the V1V2 data set due to suspicion of index swap (a frequent occurrence in water extracts).

^d^ Consensus in V3V4 for samples SCC+cfu+ and SCC+cfu−.

^e^ Found in the V3V4 run of all sample prototypes but without consensus between extraction protocols.

^f^ Consensus in V1V2; found with V3V4 in all samples but without consensus between extraction protocols.

^g^ Consensus in V1V2, except sample SCC+cfu−; consensus in V3V4 for sample SCC+cfu+ and SCC+cfu−.

^h^ Consensus in V3V4 for samples SCC−cfu− and SCC−cfu+.

^i^ Consensus in V1V2; the consensus in V3V4, except for sample SCC−cfu−.

^j^ Consensus in V3V4 only for sample SCC−cfu+.

^k^ Consensus in V1V2, except for sample SCC+cfu−.

^l^ Consensus in V1V2 only for sample SCC+cfu+.

**Table A4:** *p* Values for α‐diversity indices^a^ between the resequenced milk samples^b^ within the DNA extraction protocols (P3: QIAamp DNA Mini kit; P4, P6: modified DNeasy Blood and Tissue kit)

	Shannon		Richness		Evenness	
Protocol	V1V2	V3V4	V1V2	V3V4	V1V2	V3V4
P3	0.21	**0.013**	0.16	**0.00027**	0.37	0.92
P4	**0.0018**	**0.047**	0.065	**0.0011**	**0.00005**	**0.016**
P6	**0.0053**	**0.00034**	**0.0034**	**0.0063**	**0.029**	**0.00066**

*Note*: Bold indicates significance at *p* < 0.05. All samples were extracted in two (P4) or three replicates (P3, P6) and sequenced in triplicates.

Abbreviations: ANOVA, analysis of variance; cfu, colony‐forming unit; SCC, somatic cell count.

^a^ α‐Diversity indices Shannon, richness, and evenness with primer pairs V1V2 and V3V4 are shown by applying an ANOVA.

^b^ Prototype samples include unsuspicious milk (SCC−cfu−), milk suspect of unspecific (SCC+cfu−; SCC+cfu+), or “latent” infection (SCC−cfu+).

Figures A4 (V1V2) and A5 (V3V4) show the distribution pattern of the bacterial genera as well as the clustering of the resequenced milk samples SCC−cfu−, SCC+cfu−, SCC+cfu+, and SCC−cfu+. The three selected DNA extraction kits P3, P4, and P6 are compared. In the sequencing runs, the milk extracts clustered into two (V1V2) and three (V3V4) branches (groups) characterized by different taxonomic compositions. One group consisting of 60 (66%) samples at V1V2 and 53 (59.6%) samples at V3V4 was characterized by a high abundance of *Corynebacterium*, *Staphylococcus*, and *Streptococcus*. The second group in the V1V2 run, consisting of 31 (34%) samples also showed proportions of *Corynebacterium* and *Staphylococcus*, but in lower amounts. The group was dominated by the taxa of *Vibrio* and *Propionibacterium*, unclassified kingdoms, and unclassified *Proteobacteria*. The second group in the V3V4 run, consisting of 11 (12.4%) samples, was mostly composed of taxa of unclassified bacteria, *Acinetobacter* and *Sphingomonas*. A majority of the sample SCC−cfu+ clustered in the third group consisting of 25 samples (28%). Here, *Corynebacterium* and *Staphylococcus* were mainly predominant. The sequencing replicates of the extractions clustered for the majority of the samples, as can be seen, for example, in SCC+cfu− and SCC+cfu+ (P4) as well as in SCC−cfu− (P6) in Figure 3 (V1V2) or SCC−cfu+ (P3, P4). However, with P6, one extraction replicate of sample SCC−cfu+ was placed in another branch than the other two replicates and most of the sequence sets were gained with the other kits.
